# Cryptosporidiosis among Hemodialysis Patients in Jordan: First Preliminary Screening Surveillance

**DOI:** 10.3390/tropicalmed4040131

**Published:** 2019-10-18

**Authors:** AbdelRahman M. Zueter, Nawal S. Hijjawi, Khaled N. Hamadeneh, Maysa M. Al-Sheyab, Amal M. Hatamleh

**Affiliations:** 1 Department of Medical Laboratory Sciences, The Hashemite University, Zarqa 13133, Jordan; nhijjawi@hu.edu.jo; 2 Nephrology Department, King Hussein Medical Center, Amman 11118, Jordan; KhaledH@rms.jo; 3 Medical Microbiology Department, Prince Rashed Bin AL-Hassan Military Hospital, Irbid 21110, Jordan; Meso@rms.jo; 4 Medical Hematology Department, Prince Rashed Bin AL-Hassan Military Hospital, Irbid 21110, Jordan; AmalMufade@rms.jo

**Keywords:** cryptosporidiosis, hemodialysis, Jordan, acid-fast stain

## Abstract

Few studies have reported the incidence of cryptosporidiosis among hemodialysis patients worldwide. Currently many molecular and immunological assays have been developed for the sensitive diagnosis of cryptosporidiosis, but still, the microscopic detection of the parasitic infective stage (oocysts) in stool specimens using modified acid stain is regarded as a reliable sensitive technique which is widely used in many clinical labs. In the present study, a total of 133 stool samples were collected from hemodialysis patients and were screened for *Cryptosporidium* oocyst using formalin-ether concentration and modified acid-fast staining technique. Clinical and demographic data were also collected and analyzed. *Cryptosporidium* oocysts were recovered in 15/133 (11%) of the investigated hemodialysis patients. The age of patients ranged from 25 to 80 years (mean: 57.84 ± 12.22). Most of the *Cryptosporidium*-positive cases were recovered from males (73.7%) residing in rural villages in Irbid city (86.6%). The most repeatedly reported symptoms in the *Cryptosporidium*-positive patients were gastrointestinal symptoms, including diarrhea (15%), nausea (24%), abdominal pain (23%) and bloating (17%), in addition to general fatigue (32%) and weight loss (19%). No statistically significant associations for certain clinical symptoms or risk factors were found. The present study is the first preliminary study in Jordan that provided a brief screening for the incidence of cryptosporidiosis among hemodialysis patients.

## 1. Introduction 

*Cryptosporidium* species is an important intracellular apicomplexan protozoan parasite that infects a wide range of hosts including humans, domestic and wild animals worldwide [1,2]. *Cryptosporidium* has been ranked as the sixth most important food-borne parasite worldwide [3].

Human cryptosporidiosis was first observed in 1976, and it was later recognized as a major cause of chronic diarrhea in AIDS patients and children and was responsible for many zoonotic and waterborne diarrheal outbreaks worldwide [4]. Furthermore, awareness of cryptosporidiosis was growing globally, especially among malnourished children and reported to be a cause for premature death in low-resource settings [5,6,7]. 

*Cryptosporidium* life cycle starts upon the ingestion of the oocysts that shed with the infected host’s stool and can be transmitted to new hosts via the fecal–oral route [8]. Cryptosporidiosis is usually self-resolving in immunocompetent individuals; however, it might lead to life-threatening complications in immunocompromised patients [2]. Cryptosporidiosis causes acute diarrhea with elevated morbidity and mortality in children, immunocompromised patients, such as AIDS patients, cancer patients and hemodialysis patients [3,9,10,11]. 

Microscopy using acid-fast staining, with or without stool concentration, is the most widely used screening technique globally for the diagnosis of *Cryptosporidium* since it is cheap and accessible especially in rural settings and shows high sensitivity in comparison with other parasitological, immunological and even molecular techniques [2,10,12].

*Cryptosporidium* oocysts were investigated in several studies either alone or along with other intestinal parasites among different population groups [13,14,15,16], as well as, in the environment [17,18,19] and domestic animals [20,21]. In Jordan, studies on *Cryptosporidium* were conducted on normal and immunocompromised patients among different age groups [10,12,22,23,24,25]. 

To increase the awareness toward *Cryptosporidium* infection in Jordan, hemodialysis patients were included in this report. Hemodialysis patients usually suffer from frequent episodes of diarrhea and various gastrointestinal symptoms, and thus suspected to be at an increased probability of shedding a hidden cryptosporidiosis which might be unpredicted by the treating physician since the infection is neglected among these patients and has never been investigated before [10].

Additionally, hemodialysis patients are known to experience immune suppression, so they are at high risk of acquiring cryptosporidiosis more than any other patient [26]. Moreover, hemodialysis patients usually suffer from chronic renal failure since they have defects in kidney functions that lead to retention of uremic toxins in the blood. Uremic toxins interfere with cellular and humoral immunity functions leading to increased individual susceptibility to infections [26]. Uremic toxins-associated immune deficiency is considered as the second most significant factor responsible for the high morbidity and mortality among chronic renal failure patients after cardiovascular diseases [27]. 

Parasitic infections are one of the significant causes of morbidity and mortality in hemodialysis patients [26]. Intestinal parasites, in particular, Entamoeba histolytica, Giardia lamblia, opportunistic *Cryptosporidium* species and Strongyloides stercoralis are prevalent in developing countries, especially among chronic kidney disease patients who are undergoing hemodialysis [26,28]. 

A few studies performed in several countries had reported the incidence of cryptosporidiosis among hemodialysis patients and had applied mainly modified acid-fast staining microscopy as the screening diagnostic method of choice [26,29,30]. Therefore, the main aim of the present study was to provide a preliminary investigation for the incidence of cryptosporidiosis among hemodialysis patients in Jordan using simple modified acid-fast staining. 

## 2. Materials and Methods

### 2.1. Patients Recruitment and Ethical Approval 

This study was done after obtaining ethical approval from the institutional review board (IRB) at Hashemite University (number 16/11/1800857). Informed consent was obtained from all participants (hemodialysis patients and normal individuals who served as a negative control).

In the present study, a total of 133 fresh stool samples were collected from hemodialysis patients at King Hussein Medical Center in Amman and Prince Rashed Bin AL-Hassan Military Hospital, Irbid from April to October 2018. At the same time, 50 fresh stool samples were collected from healthy individuals from the same collection places to serve as a negative control.

### 2.2. Demographic and Clinical Data Collection

Recruited hemodialysis patients in this study belong predominantly to low-income communities. All patients were orally consented to participate in this study by the staff nurse and they answered a standardized questionnaire concerning demographic data, such as sex, age, residency and symptoms related to intestinal manifestations such as diarrhea (having at least 3 loose stools a day), abdominal pain (diffuse pain in the abdominal cavity), stomach pain (cramps in the tummy), weight loss (decrease >5% of body weight within 6 months), fatigue (sustained decrease in the strength), nausea or vomiting (frequent sensation or acting of forcible emptying of the stomach) and bloating (abnormal stomach swelling or feeling full). Data analysis was carried out using the SPSS software version 25 (SPSS, Chicago, IL, USA).

### 2.3. Stool Samples Testing

Collected stool samples were preserved in 10% formalin and kept in a cool and dry place until examination. Stool samples were concentrated and stained by a modified acid-fast staining technique before being screened for *Cryptosporidium* oocysts, as described previously [10,11]. Briefly, stool samples were concentrated using formalin-ether method and a separate thin microscopic smear was prepared for each sample. The smears were then fixed with absolute methanol for 30 seconds. Thereafter, smears were stained with carbol fuchsin for 3 minutes, rinsed briefly with tap water, decolorized by 3% acid alcohol for 4 seconds and then counterstained with methylene blue for 1 minute. The stained dried slides were labeled and examined for the presence of *Cryptosporidium* oocysts at an oil immersion lens under a light microscope.

## 3. Results

Clinical and demographic data were recovered for 100 out of 133 patient’s files and were reviewed. In the present study, the age of patients ranged from 25 to 80 years (mean: 57.84 ± 12.22) and more than two-thirds of them were males 72% (72/100). Microscopic screening of the 133 stool specimens in the present study identified *Cryptosporidium* oocysts in 15 hemodialysis patients (11%) and no oocysts were observed in any of the control samples (see Table 1).

During the present study, more than half of stool samples were collected from patients living in Irbid city in Northern Jordan, while the remaining samples were collected from residents in other cities of Jordan such as Amman, Al-Salt, Jerash, Madaba, and Zarqa. Thirteen out of fifteen (86.6%) *Cryptosporidium*-positive patients came from rural villages in Irbid.

Most *Cryptosporidium*-positive hemodialysis patients were experiencing at least one of the investigated gastrointestinal symptoms, including diarrhea 15%, nausea 24%, abdominal pain 23% and bloating 17%, in addition to general fatigue 32% and weight loss 19%. However, no statistically significant (p-value > 0.05) association for certain clinical symptoms or risk factors was reported during the present study (data not shown).

## 4. Discussion

Hemodialysis patients are more susceptible to opportunistic parasitic infections since their immune response is impaired [26]. The incidence rate of cryptosporidiosis among hemodialysis patients is severely underestimated worldwide since few studies have been performed in different countries [31]. In Jordan, no studies to assess the prevalence of cryptosporidiosis among hemodialysis patients were performed. Regarding *Cryptosporidium* prevalence among patients with impaired immune response, only one single study was performed to assess the prevalence of cryptosporidiosis among pediatric oncology patients which reported a higher prevalence (14.4%) compared to pediatric non-oncology children (5.1%) [11]. The above-mentioned study had encouraged further investigations for cryptosporidiosis infection rates among targeted patients’ groups such as hemodialysis patients who are exposed to episodes of immunosuppression. 

In the present study, the prevalence of cryptosporidiosis among hemodialysis patients was 11% and the age of patients was ranged from 25 to 80 years (mean: 57.84 ± 12.22) with more positivity seen among males 72% (72/100). The percentages observed in this study harmonized with those obtained from similar studies worldwide [30,31,32] and with results obtained previously in Jordan from pediatric cancer patients 14.4% [10]. However, the prevalence of cryptosporidiosis in Jordan ranges from 4 to 19% and modified acid-fast staining microscopy was frequently used as the diagnostic method of choice [10,12,24,33]. Moreover, the prevalence of cryptosporidiosis in Jordan was investigated in many studies on different settings (elementary school children 4%, in pediatric cancer patients 14.4%, in preschool children 6.7%, in patients with diarrhea living in rural areas 8% and in children 19% [10,24,25,33,34]. Based on the literature, the incidence of hemodialysis in Jordan is common among male gender [35], which explains the increased cryptosporidiosis positivity among males in this study; 11 males versus 4 females (see Table 1). 

More than half of the stool samples were collected from patients living in Irbid city in Northern Jordan and 86.6% of *Cryptosporidium*-positive patients came from rural villages in Irbid. This came in agreement with other conduced previous studies in Jordan which reported high incidence rates of cryptosporidiosis among populations of rural areas of Irbid city [12,22,25]. Several risk factors attributed to the increase in the cryptosporidiosis infection were in Northern Jordan, such as the use of underground water for drinking which is usually exposed for contamination from the sewage, contact with domestic animals, and eating unwashed vegetables [22,24,33]. Also, it was reported that the cryptosporidiosis rate in the rural population was higher than the urban population in the hemodialysis patients’ group [11,26].

Gastrointestinal symptoms were seen in most *Cryptosporidium*-positive hemodialysis patients with neither predomination of certain symptoms nor statistically significant association for certain clinical symptoms or risk factors. In general, several studies reported similar demographical and clinical findings among hemodialysis patients in many countries [11,26,31,36]. Diversity in prevalence rates of cryptosporidiosis among hemodialysis patients is attributed to the prevalence of parasitic infection in the region of sampling, environmental, climatic and sanitary factors [26]. Table 2 summarizes recent updates regarding cryptosporidiosis prevalence among hemodialysis patients in different countries. 

## 5. Conclusions

This is the first preliminary study in Jordan that provided brief screening for the incidence of cryptosporidiosis in hemodialysis patients. More clinical and molecular epidemiological surveys should be conducted in Jordan on the immunodeficient hosts such as hemodialysis and cancer patients to provide a better understanding of *Cryptosporidium* prevalence, pathogenesis, and routes of transmission among this category of patients. 

## Figures and Tables

**Table 1:** Positive cryptosporidiosis among gender.

	Modified Acid-Fast Microscopy		
Gender	Negative	Positive	Total
Female	24	4 (26.3%)	28
Male	61	11 (72.7%)	72

**Table 2:** Cryptosporidiosis among hemodialysis patients worldwide.

Country	Study Interval	Sample Size	Prevalence	Risk Factors	Diagnostic Method	Ref
Egypt	Aug. 2014–Apr. 2015	330	3%	Residency, hygiene, education	Concentration and modified Ziehl–Neelsen staining	[11]
Egypt	Jan.–Dec. 2017	150	40%	Contact with domestic animals	Wet mount, Concentration and modified Ziehl–Neelsen staining	[26]
Brazil	Apr.–Sept. 1993	78	10%	Not reported	Concentration and modified Ziehl–Neelsen staining	[32]
Turkey	2001	74	20%	Not reported	Modified Ziehl–Neelsen staining	[36]
Iran	2000–2001	104	11.5%	Diabetes	Modified Ziehl–Neelsen staining	[31]
Brazil	Apr. 2006–Sept. 2007	86	4.7%	Middle-age group	Kinyoun acid-fast staining	[29]
Iran	May 2013–Jan. 2014	78	11.5%	Residency	Direct-smear, formol-ether and modified Ziehl–Neelsen staining	[30]
Iran	1997–1999	103	3.88%	Duration of hemodialysis	Modified Ziehl–Neelsen staining	[37]
